# The Role of Extended Reality in Orthodontic Treatment Planning and Simulation-A scoping Review

**DOI:** 10.1016/j.identj.2025.103855

**Published:** 2025-08-29

**Authors:** Anand Marya, Siddharthan Selvaraj, Katsushi Okazaki, Ding-Han Wang, Hiroyasu Kanetaka, Thantrira Porntaveetus

**Affiliations:** a Department of Orthodontics, Faculty of Dentistry, University of Puthisastra, Phnom Penh, Cambodia; b Faculty of Dentistry, University of Puthisastra, Phnom Penh, Cambodia; c Department of Endodontics, New York University College of Dentistry, New York, NY; d Department of Endodontics, Tokyo Dental College, 2-9-18, Kanda-Misakichou, Chiyoda-ku, Tokyo 101-0061, Japan; e National Yang Ming Chiao Tung University, College of Dentistry, Taipei, Taiwan; f Division of Orthodontics and Dentofacial Orthopedics, Tohoku University Graduate School of Dentistry, Sendai, Japan; g Center of Excellence in Precision Medicine and Digital Health, Geriatric Dentistry and Special Patients Care International Program, Department of Physiology, Faculty of Dentistry, Chulalongkorn University, Bangkok Thailand; h Clinic of General-, Special Care and Geriatric Dentistry, Center for Dental Medicine, University of Zurich, Zurich, Switzerland

**Keywords:** Augmented reality, Dental education, Extended reality, E-learning environment, Patient communication, simulation, Health care access, Virtual reality

## Abstract

**Background:**

Extended reality (XR), a nomenclature covering virtual reality (VR), augmented reality (AR), and mixed reality (MR), has emerged as a breakthrough technology in dental and medical sciences. However, the scope, effectiveness, and limitations of these technologies still remain unclear.

**Aim:**

The aim of the present scoping review is to systematically outline the current state of XR applications for orthodontic treatment planning and simulation.

**Method:**

A systematic and comprehensive review of the literature was carried out in a broad array of electronic databases such as PubMed, Scopus, Google Scholar, Web of Science, Embase, IEEE Xplore, and the Cochrane Library. Studies involving the use of XR in orthodontic clinical or educational settings were included. Data extraction focused on the XR type, application purpose, target population, outcomes, and limitations. The study selection followed the Preferred Reporting Items for Systematic Reviews and Meta-Analyses extension for Scoping Reviews (PRISMA-ScR) framework.

**Result:**

A total of 19 studies were included, including VR (n = 13), AR (n = 5), and MR (n = 1) approaches. The identified applications demonstrate broad utility, ranging from clinical procedures such as orthodontic treatment planning, precision bracket placement, and complex surgical simulation, to pedagogical and patient-centered uses including immersive student training and interactive patient education. The most frequently reported positive outcomes across these diverse applications include enhanced spatial awareness, clinical accuracy, learner motivation and reductions in patient anxiety. Critically, the current evidence base, while promising, is constrained by predominantly small sample sizes across studies, significant methodological heterogeneity hindering meta-analysis, and a paucity of robust, long-term clinical outcome data.

**Conclusion:**

XR technologies hold significant promise for transforming orthodontic care and education by enhancing treatment accuracy, clinical training, and patient engagement. However, their integration into routine practice will require high-quality, large-scale studies that establish clinical effectiveness, long-term skill retention, and cost-efficiency. Generating robust evidence, including patient-reported outcomes, is essential to bridge the gap between innovation and evidence-based implementation.

## Introduction

Extended reality (XR) has emerged as a breakthrough technology in the dental and medical sciences.^1^ Among several of its applications in clinical fields, orthodontics has discovered vast potential for the application of XR in superior treatment planning, diagnostic accuracy, patient awareness and dental education. The use of XR in orthodontics is only a part of the vast process of digitization and individualized treatment in healthcare wherein immersive technologies present an interactive gateway between complex clinical information, clinicians, and patients.^2^

The XR is an umbrella term that encompasses three key immersive technologies: Virtual Reality (VR), Augmented Reality (AR), and Mixed Reality (MR). VR involves complete immersion in a computer-generated environment, often using head-mounted displays. AR overlays digital information onto the physical world using devices like smartphones or smart glasses. MR blends both, enabling real-time interaction between physical and virtual objects, often through advanced spatial mapping and responsive interfaces.^3^

Traditional orthodontic treatment planning involves a formal analysis with physical models, cephalometric radiographs, and two-dimensional images.^4^ Although effective, there are limitations in visualizing complicated craniofacial anatomy and simulating the effects of treatment in an exhaustive and interactive manner. XR technologies surpass these limitations by providing immersive three-dimensional visualization, real-time manipulation of anatomical models, and simulation environments that enable clinicians to manipulate and explore a variety of treatment cases.^5^ By these immersive capabilities, XR not only provides a better understanding of patient-specific anatomy but also enables more precise treatment planning and delivery.

The VR, which transports the user to a fully computer-generated environment, has been utilized in orthodontics for both education and clinical applications. For example, wire bending operations, appliance adjustment, and bracket placement with realistic haptic feedback and interactive modules are being taught to dental students through VR-based simulators.^6^ The AR, however, superimposes digital information onto the real-world environment, augmenting the field of view of the orthodontist during the treatment. AR technology such as head-mounted displays or smartphone applications enables clinicians to demonstrate cephalometric measurements or bracket placement over the patient's dentition directly and in real time.^7^ The MR, with the aspects of both VR and AR, does much more as it has real-time interaction between the real and virtual environments, enabling collaborative planning and improved patient-physician interaction.

The XR technologies also allow for more accurate orthodontic simulations. Digital treatment planning software has existed for a long time to perform simulation of tooth movement, but XR does this better by showing it in 3D space and allowing users to see it from different angles and simulate force dynamics.^8^ XR allows for better visualization of spatial relationships between craniofacial structures and thus allows for prediction of potential complications and adjustment of treatment plans. In orthognathic surgery, for example, AR is used to superimpose surgical plans over the patient's anatomy before actual surgery, reducing intraoperative errors and maximizing outcomes.^9^

Another critical field in which XR is engaged is patient education and patient engagement. Success with orthodontic treatment often hinges on patient compliance, especially in prolonged treatment involving aligners, retainers, or elastics.^2^ Evidence shows that XR technologies facilitate comprehension of complex procedures by enabling patients to visualize their dental anatomy and even potential treatment outcomes. VR treatment pathway simulations not only increase patient comprehension but also alleviate anxiety and boost confidence in clinical decision-making. By actively involving patients in the treatment planning process, XR facilitates collaborative decision-making and can enhance compliance with prescribed therapeutic protocols.^10^

Several studies have explored the potential of XR technologies to enhance educational outcomes, particularly in orthodontics.^11^, ^12^, ^13^, ^14^, ^15^, ^16^ A comprehensive study conducted by Rao GKL et al.^17^ revealed AR simulation technology requires incorporation into e-learning pedagogy. AR visual and haptic cues can be integrated to facilitate the acquisition of the critical skill of bracket placement on teeth, hence increasing the psychomotor and cognitive skill development of students. The technique seeks to enhance the student's confidence to execute the necessary task on a real patient by providing training in an AR environment without the presence of a patient. Similarly, VR simulators have also demonstrated their efficacy in preclinical training environments, particularly by reducing the learning curve for new students and enhancing their psychomotor skills.^18^ The wise application of XR technologies in orthodontic education goes a long way in not only improving the overall quality of the learning experience but also effectively addresses the serious issues that arise due to limited patient interaction during the initial stages of clinical training.

Furthermore, XR has also been beneficial in maximizing digital workflows in orthodontic practices. With increasing usage of intraoral scanners and 3D imaging technology, XR provides a perfect platform for viewing and manipulating data.^19^ The superimposing Cone-Beam Computed Tomography (CBCT) data with AR interfaces helps clinicians to view cortical bone thickness, root anatomy, and maxillary sinuses, which are critical in procedures such as temporary anchorage device (TAD) placement.^20^ Such enhanced visualization reduces complications and maximizes biomechanical planning accuracy.

The field of XR is an exciting new frontier for orthodontic simulation and treatment planning. Its interactive and immersive nature facilitates the provision of improved diagnostic accuracy, better educational results, increased patient engagement, and enhanced interdisciplinary collaboration. However, to achieve its full potential, there must be continued research, the development of standardized protocols, and compatibility with established clinical paradigms. The aim of the present scoping review is to systematically outline the current state of XR applications for orthodontic treatment planning and simulation. The reason for performing the review is owing to the observed increase in digital technologies used in orthodontics and the need to systematically review the role played by XR in enabling this transformation.

## Methodology

This scoping review was conducted using the Joanna Briggs Institute (JBI) methodology framework recommended for scoping reviews and adhered to the PRISMA-ScR (Preferred Reporting Items for Systematic Reviews and Meta-Analyses extension for Scoping Reviews) checklist recommended by Tricco et al.^21^

### Identifying the research question

The main objective of this scoping review was to systematically map the existing literature on the role of XR including VR, AR, and MR in orthodontic treatment planning and simulation. The central research questions were:

1. What types of extended reality technologies have been used in orthodontic planning and simulation?
2. In what ways have these technologies impacted clinical decision-making, patient outcomes, and educational purposes in orthodontics?
3. What gaps exist in the literature that warrant further exploration?

The Population–Concept–Context (PCC) framework was used to define the eligibility criteria:

1. Population: Orthodontic patients, clinicians, and students.
2. Concept: Extended reality (AR, VR, MR) applications in treatment planning and simulation.
3. Context: Clinical, educational, and research settings in orthodontics.

### Identifying relevant studies

To ensure a comprehensive and systematic identification of relevant studies, an extensive search was carried out across several academic databases, including PubMed, Scopus, Web of Science, Embase, IEEE Xplore, and the Cochrane Library. Grey literature was also explored using Google Scholar (screening the first 200 results) and ProQuest Dissertations & Theses Global, with the aim of identifying potentially non-indexed or unpublished studies. The search strategy was developed in consultation with academic librarians and field experts to ensure methodological rigor. It incorporated a combination of controlled vocabulary terms—such as Medical Subject Headings (MeSH) in PubMed and EMTREE terms in Embase—alongside synonyms and related concepts, including “VR,” “AR,” and “immersive technology,” “simulation,” and “orthodontic education.” Boolean operators (“AND” “OR”), phrase searching, truncation symbols, and field tags were systematically applied to improve both precision and sensitivity of results. The final query strings included combinations such as (“Extended Reality” OR “Virtual Reality” OR “Augmented Reality” OR “Mixed Reality” OR “XR” OR “Immersive Technology”) AND (“Orthodontics” OR “Orthodontic Training” OR “Orthodontic Simulation” OR “Dental Education” OR “Orthognathic Surgery Planning”). The search was limited to peer-reviewed articles, conference proceedings, and dissertations published in English between January 2010 and March 2025, thereby focusing on research relevant to contemporary and emerging XR applications in orthodontics (Table 1).

**Table 1:** An overview of the search strategies and results.

Database	Search strategies	Results
PubMed	("Virtual Reality"[MeSH] OR "Augmented Reality"[MeSH] OR "Mixed Reality" OR "XR" OR "Extended Reality") AND ("Orthodontics"[MeSH] OR "Dental Simulation")	210
Scopus	TITLE-ABS-KEY("Extended Reality" OR "Virtual Reality" OR "Augmented Reality") AND TITLE-ABS-KEY("Orthodontics" OR "Dental Education" OR "Simulation")	185
Web of Science	TS=(“Virtual Reality” OR “Augmented Reality” OR “XR”) AND TS=(“Orthodontic Simulation” OR “Orthodontic Planning” OR “Dental Education”)	150
Google Scholar	("XR in Orthodontics" OR "AR/VR in Orthodontic Education" OR "Orthodontic Simulation XR")	260
IEEE Xplore	("XR in Orthodontics" OR "AR/VR in Orthodontic Education" OR "Orthodontic Simulation XR")	90
Cochrane Library	("Extended Reality" OR "Virtual Reality" OR "Augmented Reality") AND ("Orthodontics" OR "Dental Education")	45
ProQuest Dissertations	("Virtual Reality in Orthodontics" OR "AR Simulation in Dental Education")	55
Total		995

The complete search strategies for all databases, including Boolean operators, truncation symbols, and MeSH terms, have been appended as Supplementary Appendix A to ensure transparency and reproducibility of the review process.

### Study selection

This scoping review was not pre-registered in OSF or PROSPERO. Given the exploratory nature of the research and the evolving scope during preliminary searches, the authors opted for iterative refinement of inclusion criteria. However, future iterations of this review will consider protocol registration to enhance transparency. All the identified citations were imported into Covidence, a review management software, and duplicates were automatically removed. Screening was conducted in two phases: initial title and abstract screening and full-text assessment. Two independent reviewers conducted both screening phases in accordance with pre-set inclusion and exclusion criteria. Included studies were those that described or evaluated the use of XR technologies in orthodontic diagnosis, planning, treatment simulation, or education simulations, or education environments directly relevant to orthodontics. The types of studies that may be included in the study are randomized controlled trials, cohort and cross-sectional studies, and qualitative studies. Studies were included if they explicitly focused on XR applications in orthodontics. Mixed-scope studies (e.g., general dental education) were only included when orthodontic relevance was clearly articulated in study objectives, methods, or results. Studies with ambiguous or minimal orthodontic contexts were excluded to maintain scope specificity. The exclusion criteria were studies on general dentistry or nonorthodontic uses alone, papers that were not related to XR or simulation, opinion articles lacking empirical data, case reports, review articles, and publications not in the English language. Discrepancies at screening were solved through discussion or reference to a third reviewer (NT). Selection was documented through a PRISMA flow diagram, reporting the number of screened records, included and excluded, and the reason for exclusion at each point.

### Charting the data

Data extraction was facilitated by an iteratively developed and updated pre-designed form of data charting by the review team. Pilot testing with a subset of studies enabled optimization for clarity and consistency. Two reviewers independently extracted the data, with discrepancy resolution by consensus. Extracted data were the following: authorship, year of publication, country of origin, study design, sample characteristics, type of extended reality technology employed (AR, VR, or MR), device or software used, the purpose of the application targeted, target population, key results, limitations, and conclusions.

Semi-structured interviews were conducted with five domain experts, including orthodontists and dental educators, to explore stakeholder perspectives on the implementation of XR technologies. The thematic analysis of the interview data revealed several key insights. First, usability concerns were highlighted, particularly discomfort associated with prolonged headset use and the need for adequate user training. Secondly, significant implementation barriers were identified, such as the high cost of XR systems, hardware limitations, and challenges related to integrating XR platforms with existing orthodontic software. Finally, the experts emphasized the lack of standardized clinical protocols and robust evaluation frameworks as critical obstacles to the widespread adoption of XR in orthodontic practice and education.

## Result

**Fig. 1:**
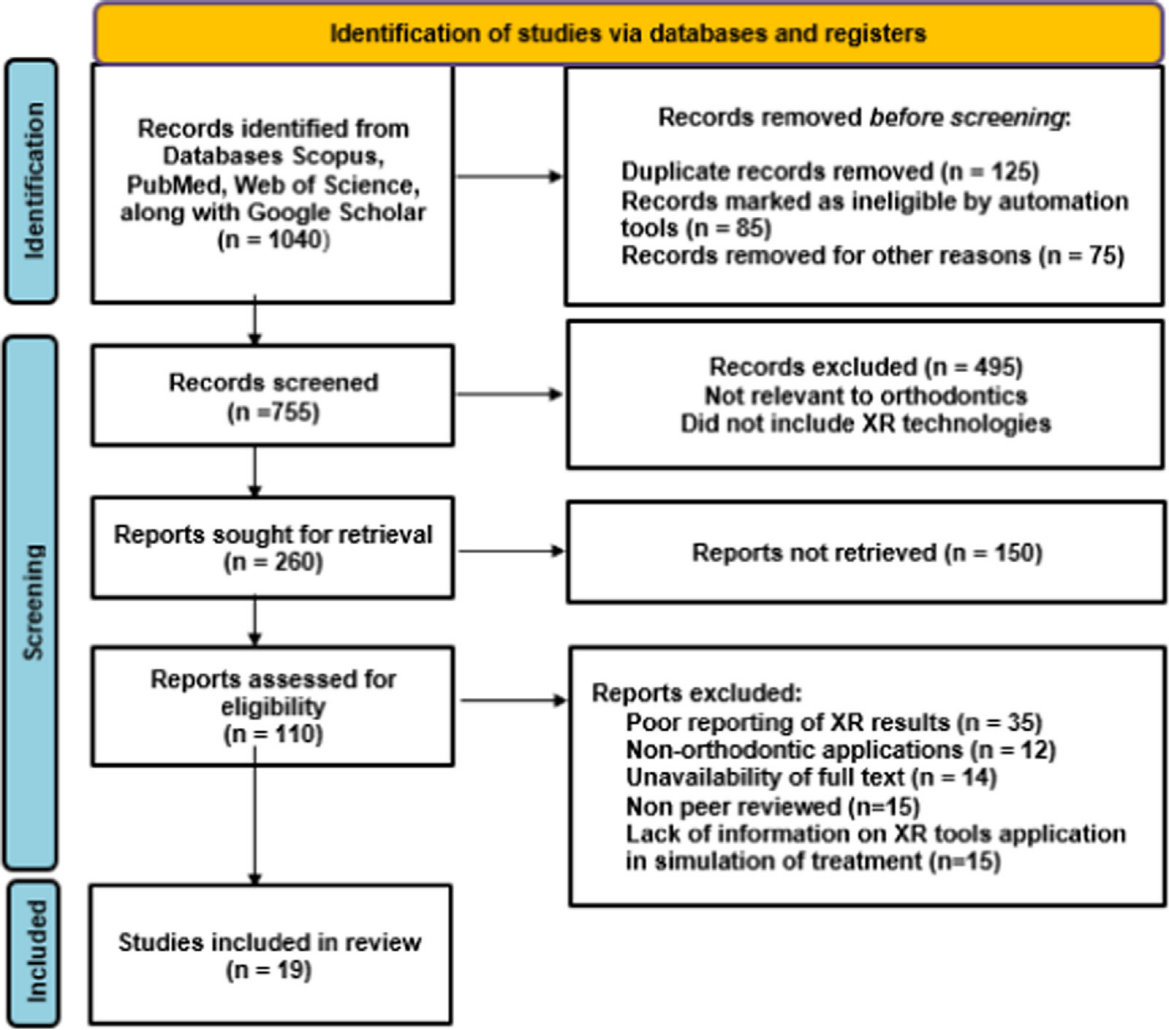
PRISMA 2020 flow diagram demonstrates the screening process of studies retrieved from different web sources.

The study selection followed the Preferred Reporting Items for Systematic Reviews and Meta-Analyses extension for Scoping Reviews (PRISMA-ScR) framework. The overall search strategy and screening are illustrated in Figure 1 (PRISMA flowchart). A systematic search conducted on seven main databases—PubMed, Scopus, Web of Science, Google Scholar, IEEE Xplore, Cochrane Library, and ProQuest Dissertations—yielded an overall 1040 articles. The studies were performed in different countries, such as Taiwan, the USA, China, Germany, Austria, the Netherlands, Spain, Iran, India, Ecuador, France, and Korea, reflecting the global interest in applying XR technologies in orthodontics.

Following the elimination of 285 duplicate records, there were 755 studies left to screen at the title and abstract level. Title and abstract screening resulted in the elimination of 495 records that were not relevant to orthodontics, did not include XR technologies, or were concerned with other unrelated topics like general dental imaging, robotic surgery, or nonsimulation-based teaching methods.

260 full-text articles were then screened for eligibility based on defined inclusion and exclusion criteria. 241 studies were excluded at this stage for a range of reasons, including poor reporting of XR results, non-orthodontic application, unavailable full text, non-peer-reviewed papers (e.g., conference abstracts or editorials), or lack of information on XR tool application in simulation or treatment planning contexts.

In total, 19 studies met all the necessary eligibility criteria and were included in the current scoping review. The studies range from 2014 to 2024 and use a variety of methodologies, including randomized controlled trials, in vitro and in vivo research, case-control studies, surveys, and educational trials.

### Types of XR technologies and devices used

Amongst the studies included and assessed in this analysis (Table 2), VR was the dominant modality used (n = 13), with AR (n = 5), and then MR (n = 1, in combination with either AR or VR). Use of these technologies was seen across a wide range of platforms and hardware, including Oculus Rift, HTC Vive, HoloLens, 3D optical scanners, CBCT, and purpose-developed VR software. AR-based research typically took advantage of intraoral scanners, CBCT scans, or smartphone-based overlays to facilitate real-time visualizations, whereas VR-based research almost universally used immersive simulation and video-based environments.

**Table 2:** Characteristics of the included studies.

Author year	Country	Study design	Sample	XR type		Device or software	Purpose of application	Target population	Key findings	Conclusion	Outcome measures used	Critical appraisal
Im J, et al.^31^	Korea	In-vitro	10		VR	3D optical laser scanner	Compare virtual and manual tooth setups	Patients	The digital setup showed smaller arch perimeters and reduced overbite, and overjet compared to the plaster model. It also displayed altered tooth angulations and inclinations. Overall, it received higher American Board of Orthodontics score deductions for overjet, occlusion, and total score.	Virtual setup comparable but requires adjustments for contact collisions.	American Board of Orthodontics Objective grading system scores	Moderate - small sample, lacks validation metrics
Müller-Hartwich R et al.^20^	Germany	In-vitro	26		VR	CAD-CAM	Precision of implementing virtual setups	Patients	Median deviations were 0.19–0.21 mm for translation and 1.77°–3.04° for rotation, with accuracy decreasing from front to back. Incisors demonstrated the highest precision.	Effective virtual setup execution possible with CAD-CAM systems.	Virtual matching process (three translational planes and three rotational axes)	Moderate - lacks long-term evaluation, limited generalizability
Guzmán and Ohara^32^	Ecuador	In-vitro	10		3D-VR	Orthoanalyzer software	Compare VR and conventional setup	Plaster models	Manual models showed higher values, with significant differences in six accuracy measures, one intra-arch, and all inter-arch parameters. Maxillary models had more variation than mandibular.	Three-dimensional setups produced using fused deposition modeling differ significantly from conventional setups.	Printed and manual setups were measured to compare dimensional accuracy and inter- and intra-arch characteristics.	Weak - in-vitro design limits clinical relevance
Sakowitz SM et al.^30^	USA	Randomised Controlled Trial	30		VR	Oculus VR headset	Diagnosis and treatment planning of orthognathic surgery	Students	MCQ scores improved post-intervention, but no group differences were found in any assessments. Effect sizes varied (.14–.90), with high scoring reliability (> .928).	Dental students improved their understanding of orthognathic case planning using both 2D tracing and VR methods. The reliable scoring method is suitable for future large-scale studies.	Dependent variables included MCQ exams, baseline and exit surveys, and written case analyses. Student–teacher interactions were also recorded by length and type.	Strong - RCT design, but limited to subjective outcome
Baan F, et al.^19^	Netherlands	In-vitro	10		VR	CBCT	Clinical precision of virtual orthodontic configurations	Patients	Observers showed high agreement, with minor translational (≤0.45 mm) and larger rotational differences, especially in molar pitch (10.26°). Upper teeth exhibited excessive extrusion and forward movement; lower molars extruded less.	Virtual setups are feasible and detailed; new insights achievable.	Differences between the virtual setup and final outcome were recorded, along with inter-observer agreement using ICC.	Moderate - small sample size, but robust outcome reporting
Ye F, et al.^33^	China	In-vitro	1		VR	Force feedback devices	Bracket placement training	Hardware	A virtual system was developed to simulate the full orthodontic bracket bonding process, including auxiliary steps like smearing, etching, and washing.	Useful for customized case training and bracket placement.	The haptic thread uses an octree-based algorithm to simulate multi-point interactions between tools and the virtual environment.	Weak - low sample data, limited validation
Sytek L et al.^34^	USA	Mixed-method study	20		VR	2D and 3D simulations	Planning orthognathic surgery	Students	More surgical movements were prescribed using 3D and VR methods (p = 0.001), though treatment plan scores were similar across all tasks. VR and 3D tasks took longer (p < 0.001) and prompted more questions about tool use and features (p < 0.002).	Results show that higher-fidelity simulation methods (3D and VR), when used with traditional records, are effective alternatives to conventional 2D orthognathic surgical simulations.	Attitudinal responses were collected through a post-survey and interview following the simulation experience.	Moderate - mixed method adds depth, but small size
Lo Y-C et al.^6^	Taiwan	In-vivo	4		AR	Scanner + software	Bracket positioning accuracy	Patients	AR-assisted bonding showed significantly lower vertical deviation than the control for both novice and expert orthodontists (p < 0.05).	The AR-assisted system improved expert accuracy in the incisogingival direction and helped novices place brackets within a 0.5 mm clinical error margin.	Bracket placement accuracy and positional deviations were measured.	Weak - extremely small sample limits conclusion
Goldani Moghadam M et al.^26^	Iran	In-vitro	66		VR	Videos	Reduce anxiety during dental treatment	Patients	The Mann-Whitney U test showed a significant difference in anxiety levels between experimental and control groups (P = .014). However, no significant correlations were found between anxiety scores and age or gender in either group.	VR effective in minimizing treatment-related anxiety.	The Beck Anxiety Inventory (BAI) was used to assess participants' anxiety levels before and after treatment.	Strong - decent sample size and validated instrument
Gredes T, et al.^27^	Germany	Survey	127		AR	3D Model scanner	Manufacture of removable orthodontic device	Students	Yearly Likert scores and confidence intervals indicated strong positive feedback on the app as a teaching tool. However, students preferred a blended approach, combining digital tools with physical plaster casts for optimal skill development.	The survey shows a generally positive attitude toward computer-assisted simulations as effective tools for learning and skill development in a multimedia environment.	Yearly mean Likert scores	AR-Demonstrator-App's drawbacks were concealed. App improvement should be done.
Bindhani BR et al.^25^	India	Case-control	70		VR	VR headgear	Pain management during orthodontic procedures	Patients	Using virtual reality movies before and after dental treatments significantly reduced pain levels. Mean pain during treatment was 8.6 (control) vs. 6.3 (study), and post-treatment pain was 7.4 vs. 6.7, respectively.	VR beneficial for managing procedural discomfort.	Visual Analog Scale (VAS) to record the participant’s anxiety level.	Strong - clear comparison and large sample
Huang Y et al.^24^	China	Survey	82		VR	VR system	Practical skills education using VR	Students	The SUS score (76.17 ± 9.89) reflects above-average usability. Students responded positively, with males showing greater interest and skill; prior VR experience had no effect.	Promising educational tool; repeatable and cost-effective.	System usability scale (SUS), evaluation of the system's usability and maturity.	Well-rated, good performance data
Ströbele DA et al.^35^	Austria	In-vitro	4		VR	CT scan + smartphone	Bracket placement	Patient	The technique was effective and easily recognizable from all angles, supporting the potential of adopting new methods and opportunities.	AR smartphone apps show promise in bracket placement.	Smartphone for digitally planned bracket position over the patient clinical crown	Weak sample size and vague metrics
Dong J et al.^22^	China	In-vitro	4 distinct wire-bending tasks		AR	HoloLens	Wire-Bending Training System (ARAWTS)	Trainees	The proposed algorithm on the HoloLens AR device achieved high recognition accuracy with low computational complexity, confirming ARAWTS's reliability.	Reliable AR-based gesture recognition system for training.	Proposed algorithm implemented on an AR device (HoloLens) to assist wire-bending	Moderate - innovative but lacks validation
Chen D, et al.^23^	China	Randomized, cross-over	2 group		VR	PPT	Educational experience for Class II malocclusion	Students	VR slightly outperformed PPT in motivation and learning experience, with most differences having a median of 0. VR received more student recommendations, though some measures showed no significant difference between phases.	VR is a valuable supplement to traditional PPT-based education, though its benefits should not be overstated. Educators should leverage VR to address specific educational challenges.	Likert scale measured attention, relevance, confidence and satisfaction	Moderate - appropriate method but small scope
Riad Deglow E, et al.^21^	Spain	Controlled experimental trial	206		AR and VR	HoloLens, AR TOOTH	Implant placement accuracy	Patients	Significant differences were found in entry-point, end-point, and angular deviations (*P* < .001), with root perforations seen only in the FHT group and none in AR-guided groups.	AR guidance reduces complications in mini-implant placement.	CBCT and intraoral scans to measure deviation angle and horizontal angle	Strong - robust trial with large N
Chiang Y-C et al.^28^	Taiwan	In-vitro	32		VR	3D-Max	Communication tool between orthodontists and patients	Dentists and patients	The proposed system effectively enhances communication between patients and orthodontists.	Effective medium for patient-practitioner communication.	3D tool for gum morphing with tooth movement, arrangement of teeth, placement of arch-wires and brackets, and facial appearance changes.	Moderate - lacks comparative outcome
Hsu M-C et al.^18^	Austria	In-vitro	2		AR	CBCT	Miniscrew placement accuracy	Patients	The AR system improved miniscrew placement accuracy for both senior and junior clinicians, with juniors showing greater gains and lower placement error.	AR improves precision regardless of clinician experience.	CBCT and AR-aided system for position and angle of miniscrew placement	Weak - extremely small sample, limited value
Bouhlal I, et al.^29^	France	In-vitro	122		VR	Lumeen VR headset	Reduce pain and anxiety during debonding	Patients	Pain/anxiety reduced; first study to use VR for debonding.	VR effective distraction during orthodontic appliance removal.	Anxiety before debonding and their perception of pain.	Strong - high sample and validated scales

### Clinical applications of XR in orthodontic practice

The majority of studies (n = 10) involved clinical treatment planning and simulation of procedures. The use included bracket placement, miniscrew placement, orthodontic setup planning, and communication of orthognathic surgical movement. In studies like Lo et al.^7^ and Hsu et al.^22^ AR-based bracket navigation and miniscrew placement aids were demonstrated to improve accuracy significantly, especially in inexperienced clinicians. Baan et al.^23^ and Müller-Hartwich et al.^24^ ascertained virtual orthodontic setup translational accuracy, and Riad Deglow et al.^25^ illustrated the reduction of root perforation rates with the use of AR tools during implant placement.

### Educational and training use of XR in orthodontics

Twelve studies have reported the use of XR in orthodontic education and training for the purposes of improving students' psychomotor skills, diagnostic reasoning, and spatial awareness. The simulations ranged from width, i.e., bracket bonding and wire bending^26^ to extensive treatment simulation and case-based learning methodologies.^27^ Studies consistently indicated that VR-based learning enhanced the engagement and satisfaction of students. For example, Huang et al.^28^ indicated a high usability score (SUS = 76.17), reflecting high user acceptance. Similarly, Bindhani et al.^29^ and Goldani Moghadam et al.^30^ demonstrated that VR significantly reduced dental anxiety and enhanced clinical performance among trainees. However, some students, according to Gredes et al.^31^ favoured mixed models of learning combining physical models and computer simulations.

### XR in enhancing patient communication and experience

Five studies examined the impact of XR on patient emotional experience and communication throughout orthodontic treatment. XR technology was used to visualize tooth movement, simulate treatment outcomes, and improve doctor-patient communication. Chiang et al.^32^ designed a VR tool where orthodontists could manipulate 3D tooth models to explain treatment procedures, receiving a high rating in usability and perceived usefulness. Bouhlal et al.^33^ and Bindhani et al.^29^ identified that VR environments significantly minimized patient anxiety and perception of pain during procedures like debonding or fixed appliance placement.

### Comparison between XR modalities and traditional methods

Most studies have compared XR-based approaches directly with control or conventional approaches. Sakowitz et al.^34^ and Im et al.^35^ found that there was no statistically significant difference in performance results between virtual reality simulation and conventional practice in diagnosis and treatment planning, but there was increased student engagement and visualization benefits with VR. Riad Deglow et al.^25^ on the other hand, presented quantitative results showing AR-guided procedures to have significantly surpassed free-hand procedures in accuracy and safety. The comparative summary matrix (Table 3) presents a side-by-side evaluation of VR, AR, and MR in relation to traditional methods across four key outcome domains: accuracy, anxiety reduction, educational engagement, and clinical feasibility. This structured comparison elucidates the unique strengths and limitations of each modality, thereby enhancing interpretability and providing actionable insights for both academic researchers and clinical practitioners.

**Table 3:** Summary matrix of XR modalities vs. traditional tools.

Outcome	VR	AR	MR	Traditional tools
Accuracy	High spatial simulation improves measurement and visualization accuracy.	Enhances real-time visualization; moderate precision boost.	Combines real-world accuracy with virtual overlays; highly precise.	Dependent on operator skill and static imaging.
Anxiety reduction	Immersive environments significantly reduce patient anxiety.	Moderate reduction via distraction techniques.	Emerging evidence for strong anxiety modulation.	Limited or no anxiety-reduction mechanisms.
Educational engagement	Highly engaging due to full immersion; effective in simulations.	Enhances engagement through interactive overlays.	Offers hybrid immersion; promising for interactive learning.	Passive, text/image-based; less engagement.
Clinical feasibility	Limited by hardware cost and setup complexity.	More feasible; compatible with mobile and head-mounted displays.	Still experimental; requires advanced hardware integration.	Highly feasible; well-established in workflows.

### Effectiveness across levels of experience

One recurring theme throughout the debate is the differential advantage that XR systems hold for junior compared to senior users. Some experiments conducted by researchers like Hsu et al.^22^ and Lo et al.^7^ revealed one interesting outcome: although senior practitioners already possess an incredibly high rate of accuracy when performing their task, it is the junior clinicians who registered most of the gains in procedural fidelity as well as in individual confidence when performing with XR support software. The finding strongly speaks to how much potential XR technology holds to serve as an integral intermediate tool for skill acquisition, considering that it lessens the burden of training on the side of early-career orthodontists, facilitating easier access to those skills and required expertise.

### Limitations noted in the literature

Though mostly positive in outcome, the studies included reported a few limitations. These include small sample sizes, short follow-up durations, and the absence of standardized XR protocols. A few simulation systems also lack realism or haptic feedback, and some reports advocate cost-benefit analysis prior to the utilization of XR technologies in routine practice. Device dependency and software complexity were also reported to be among factors that constrained its general adoption.

## Discussion

The development of orthodontics has increasingly overlapped with the introduction of digital and immersive technologies. The aim of the present scoping review is to systematically outline the current state of XR applications for orthodontic treatment planning and simulation. With the integration of findings from 19 included studies. A variety of XR technologies were used across the studies, with VR being the most commonly used modality. Devices ranged from advanced platforms like the Oculus Rift, HoloLens, and CBCT-AR implementations to more specialized educational devices based on PPT-based VR case simulations or virtual optical scanners. The diversity of hardware and software utilized spanning from commercial VR headsets to custom-made AR interfaces reflects the innovation and fragmentation characteristic of this new field.

The XR technologies are applied for diverse purposes, tailored to clinicians, trainees, and patients. VR has been widely employed for educational applications in various research studies with dental students as well as orthodontic trainees.^36^ Such studies have been carried out by researchers like Huang et al.^28^ Chen et al.^27^ and Ye et al.^37^ These researchers targeted the development of simulated environments for the purpose of training practical skills, particularly in the areas of bracket bonding and guiding students through patient case walkthroughs. AR instruments, in particular, designed from CBCT and intraoral scan data, are predominantly applied in clinical simulations to train orthodontists or in clinical accuracy studies. AR-supported systems have been utilized by Lo et al.^7^ and Hsu et al.^22^ to improve bracket placement and miniscrew placement, with real-world benefits in terms of procedural accuracy and user support, particularly for less experienced clinicians.

The reviewed studies presented significant results in support of the integration of XR into all aspects of orthodontic treatment. One of the significant effects of XR identified is enhancing spatial perception and procedural accuracy in complex clinical procedures. For example, Lo et al.^7^ demonstrated that novice orthodontists using AR-guided bracket placement systems achieved much better vertical positioning accuracy than with traditional methods. Similarly, Hsu et al.^22^ showed that AR reduced positional and angular errors significantly in miniscrew placement for both novice and expert practitioners, providing evidence of the usefulness of XR in technical proficiency enhancement across a wide range of skill levels.

In addition to mechanical precision, XR has also contributed to the optimization of clinical treatment planning, especially in the operating room. Sakowitz et al.^34^ and Sytek et al.^38^ explored VR in orthognathic surgery planning and concluded that, although VR simulations did not substantially surpass conventional methods in written assessments, they did significantly enhance understanding and confidence levels among trainees. The qualitative feedback in the studies validated that users felt high-fidelity VR environments were more intuitive and interactive, which can positively influence decision-making and internalization of knowledge in training-to-practice transitions.

XR also plays a greater role in patient-centered care. Some studies indicate that virtual environments improve communication and patient comprehension of treatment procedures. Chiang et al.^32^ suggested a VR model with interactive functionality that enables orthodontists to demonstrate tooth movement and appliance function in a customized way, with high usability and perceived effectiveness ratings. Those findings are significant in a field where patient cooperation is essential to optimal outcomes. Similarly, three studies—Goldani Moghadam et al.^30^, Bindhani et al.^29^, and Bouhlal et al.^33^ reported VR distraction during clinical treatment like bonding or debonding decreased patient-reported pain and anxiety. These studies collectively introduce XR as not only a means to clinical excellence but also as a bridge for therapeutic engagement and emotional regulation in orthodontic treatment.^39^^,^^40^ Beyond anxiety reduction, XR also presents opportunities for enhancing shared decision-making through visualized treatment simulations. By allowing patients to see projected tooth movements or appliance mechanics, XR strengthens comprehension and supports collaborative planning. This may improve long-term adherence, treatment satisfaction, and patient empowerment dimensions that are essential in orthodontic care but insufficiently explored in current XR research.

Educationally, XR has shown a stronger and more consistent ability to improve learning outcomes, and increase motivation among dental and orthodontic education students. Specifically, extensive research by Chen et al.^27^ and Huang et al.^28^ has shed light on many of the most important advantages, such as greater learning motivation, higher perceived value of the instructional material, and increased enjoyment on the part of the students when learning in VR-based environments, particularly compared to more traditional ways through PowerPoint presentations or the traditional textbook material. Huang et al.'s The study revealed a System Usability Scale (SUS) score of 76.17 ± 9.89, indicating above-average acceptance of VR systems in training environments.^24^ Ye et al.^37^ pushed this field further by developing a fully immersive VR system for training students in the whole process of bracket placement, including auxiliary procedures like etching and washing. The hands-on nature of VR combined with its replicable and low-cost training potential makes it an inevitable tool in the digital revolution of orthodontic education. Although positive learning outcomes have been widely observed, few studies explicitly link XR benefits to educational theory. Incorporating pedagogical models such as Cognitive Load Theory (CLT) or Kolb’s Experiential Learning Cycle can contextualize why XR leads to improved skill acquisition and retention. For example, XR reduces extraneous cognitive load by spatially organizing content, while its immersive interactivity supports active experimentation—a core stage of experiential learning. Theoretical alignment would provide a more structured foundation for curriculum design and evaluation of XR tools.

From a performance improvement objective perspective, the use of XR in skill acquisition through simulation is supported by Dong et al.^26^ who demonstrated that trainees utilizing an AR-based wire-bending system with a gesture recognition algorithm had enhanced task recognition rates while minimizing computational load. Similarly, the research work carried out by Müller-Hartwich et al.^24^ in conjunction with Baan et al.^23^ also provided additional evidence that virtual environments, which utilized CAD-CAM systems in CBCT imaging, ultimately led to translational and rotational deviation values that are considered clinically acceptable. This strongly illustrates the technical viability and usability of using XR-based planning tools in real-world orthodontic workflows within a real-world setup.

Despite these reassuring outcomes, the most important limitation identified in the majority of studies was the small participant group combined with the lack of longitudinal data. Only a few studies, such as those performed by Bouhlal et al.^33^ and Riad Deglow et al.^25^ utilized large participant groups or strict controlled trial designs. Most of the rest were pilot studies, cross-sectional surveys, or in vitro studies, which limits the generalizability of their results. Moreover, while educational and psychometric outcomes are usually highlighted, few studies have tested the long-term retention of skill or the long-term effect of XR training on clinical performance. There is a pressing need for longitudinal studies and multicenter trials to establish the sustainability of XR-based learning and the stability of treatment outcomes as such technologies are implemented in clinical orthodontic practice.

Furthermore, whereas numerous investigations have focused on usability and user experience, relatively less attention has been given to cost-benefit analysis, the technology implementation barrier, and the learning curve of such platforms. The diversity of hardware platforms and software environments is the biggest hindrance to the use of standard protocols. Beyond hardware variability, implementation barriers such as high acquisition costs, lack of standardized integration with existing orthodontic software, and limited training for faculty or clinicians hinder widespread adoption. Even when XR tools are technically viable, institutional inertia, licensing constraints, and steep learning curves further reduce their practical feasibility. These findings suggest the need for implementation research to identify scalable, cost-effective deployment models in both academic and private practice settings. While some virtual reality environments use force-feedback devices to deliver tactile simulation, others use only visual immersion, thus making it difficult to compare them directly. Additionally, few investigations have examined the functioning of MR, which involves the combination of both VR and AR components, a largely untapped area of research that could facilitate an even more integrated syncretism of digital and physical spaces within the context of orthodontics. This underrepresentation of MR technologies reveals a critical research gap. Despite MR’s ability to overlay interactive 3D data onto real-world views—potentially aiding in complex tasks like multi-arch planning or real-time guided surgeries—only one study in this review utilized MR. Future investigations should explore MR's hybrid affordances in both educational and clinical workflows to determine its unique contribution beyond VR or AR alone.

Among the critical gaps in the current literature determined is inadequate representation of multivariable diverse patient populations and variables such as sex, age, and socioeconomic status, whose effects on XR usability and efficacy would likely be significant. As orthodontic treatment is patient-specific in nature, future research must establish the degree to which different patient populations interact and engage with and are impacted by XR interventions. Ethical concerns and data protection-related problems of XR systems are hardly mentioned in the studied articles. Given that XR applications frequently rely on individualized 3D health data, their use raises critical ethical, legal, and regulatory questions. Compliance with privacy frameworks such as GDPR and HIPAA is essential, especially when cloud-based or AI-enhanced tools are deployed. Moreover, XR usage in diagnostics or semi-automated planning must account for clinical accountability, patient consent in digital contexts, and the potential for algorithmic bias. These issues are notably absent from most studies, indicating a pressing need for integrative frameworks governing XR ethics and legal accountability.

### Implications for future research

The findings of this scoping review outline the directions of future research in XR technologies as applied to orthodontics. While XR technologies have been found to be effective in increasing clinical accuracy, improving educational attainment, and improving patient experience, large-scale long-term trials should be carried out to determine their long-term effectiveness and incorporation into routine clinical practice. Future research must include the creation of standard frameworks for XR implementation and the comparison of cost-effectiveness, compatibility with current digital dental systems, and clinical decision-making outcomes. Comparative effectiveness research of XR modalities (virtual reality, augmented reality, mixed reality) vs traditional approaches will be critical to guide evidence-based integration. There are also areas of potential innovation regarding the MR platform, which is still not fully realized. Research must also address user-centered design principles, accessibility, and ethical considerations of XR use, including data privacy, digital consent, and equal access for diverse patient and educational populations.

### Limitations

This scoping review had few limitations like predominance of small-scale, exploratory studies with heterogeneous methodologies and findings, which limit generalizability and preclude direct comparison. The majority of studies were on virtual reality, with fewer on augmented or mixed reality, leaving an imbalanced evidence base. Omission of non-English and grey literature might have led to the loss of pertinent findings. Finally, although educational and usability outcomes were often reported, clinical effectiveness and long-term patient outcomes were often not quantified.

## Conclusion

XR technologies are transforming the landscape of orthodontic practice and education by enabling immersive simulation, precision treatment planning, and enhanced patient communication. This scoping review has mapped the growing body of evidence demonstrating that VR and AR can improve treatment accuracy, clinical training, and patient engagement. As these technologies continue to evolve, preliminary findings are promising; however, broader clinical adoption will depend on rigorous validation, standardization, and ethical integration.

To guide future research and applications, we identify five key domains of focus. Technologically, future studies should prioritize the refinement and evaluation of MR systems with improved fidelity and interoperability. Clinically, longitudinal trials are needed to assess sustained outcomes and integrate XR into existing digital orthodontic workflows. From an educational perspective, developing standardized training modules and validated assessment metrics will ensure consistent implementation across institutions. Ethical considerations such as consent, data privacy, and user accountability must be addressed to safeguard responsible use. Finally, at the operational level, cost-effectiveness analyses, faculty development programs, and curricular reforms are essential for sustainable adoption.

## Appendix Supplementary materials

Image, application 1

## Footnotes

Supplementary material associated with this article can be found in the online version at doi:10.1016/j.identj.2025.103855.
