# Supplementary material for: The Role of Extended Reality in Orthodontic Treatment Planning and Simulation-A scoping Review
Source: Int Dent J. 2025 Aug 29;75(6):103855. doi: 10.1016/j.identj.2025.103855 (PMC12418823; doi:10.1016/j.identj.2025.103855)
Supplement: Supplementary file 1 [file mmc1.docx]

**Supplementary Appendix A**

**Complete Search Strategy with Boolean Operators and MeSH Terms**

The following search strategies were tailored to each database and conducted from their inception through March 2025.

**PubMed Search Strategy**

("Orthodontics"[Mesh] OR orthodontic* OR braces OR malocclusion)
AND ("Virtual Reality"[Mesh] OR "Augmented Reality"[Mesh] OR "Mixed Reality"[Mesh] OR "Extended Reality" OR "XR" OR "AR" OR "VR" OR "MR"
OR "immersive technology" OR "virtual simulation" OR "haptic simulation")
AND ("Education"[Mesh] OR "Treatment Planning"[Mesh] OR "Simulation Training"[Mesh] OR training OR teaching OR diagnosis OR therapy OR communication)

**Scopus Search Strategy**

(TITLE-ABS-KEY(orthodontic* OR malocclusion OR braces))
AND (TITLE-ABS-KEY("virtual reality" OR "augmented reality" OR "mixed reality"
OR "extended reality" OR XR OR VR OR AR OR MR OR "immersive technology" OR "virtual simulation" OR haptic)) AND (TITLE-ABS-KEY(education OR training OR simulation OR diagnosis OR therapy OR communication))

**Embase Search Strategy**

('orthodontics'/exp OR orthodontic*:ti,ab OR braces:ti,ab OR malocclusion:ti,ab)
AND ('virtual reality'/exp OR 'augmented reality'/exp OR 'mixed reality'/exp
OR 'extended reality':ti,ab OR XR:ti,ab OR VR:ti,ab OR AR:ti,ab OR MR:ti,ab
OR 'immersive technology':ti,ab OR 'haptic simulation':ti,ab) AND
('education'/exp OR 'training'/exp OR 'simulation'/exp OR 'treatment planning'/exp
OR training:ti,ab OR teaching:ti,ab OR diagnosis:ti,ab OR communication:ti,ab)

**Web of Science Search Strategy**

TS=(orthodontic* OR malocclusion OR braces) AND TS=("virtual reality" OR "augmented reality" OR "mixed reality" OR "extended reality" OR XR OR VR OR AR OR MR OR "immersive technology" OR "virtual simulation" OR haptic) AND
TS=(education OR training OR simulation OR diagnosis OR therapy OR communication)

**Search Filters Applied (All Databases):**

- Language: English
- Study Types: All empirical study designs (e.g., RCTs, observational, qualitative)
- Publication Status: Peer-reviewed journals
- Date: No restrictions
